# Multispecies interactions shape the transition to multicellularity

**DOI:** 10.1098/rspb.2023.1055

**Published:** 2023-09-20

**Authors:** Maria Kalambokidis, Michael Travisano

**Affiliations:** ^1^ Department of Ecology, Evolution, University of Minnesota, St. Paul, MN 55108, USA; ^2^ The BioTechnology Institute, University of Minnesota, St. Paul, MN 55108, USA; ^3^ Minnesota Center for the Philosophy of Science, University of Minnesota, Minneapolis, MN 55455, USA

**Keywords:** multicellularity, cooperation, interspecific competition, major evolutionary transitions, experimental evolution, species interactions

## Abstract

The origin of multicellularity transformed the adaptive landscape on Earth, opening diverse avenues for further innovation. The transition to multicellular life is understood as the evolution of cooperative groups which form a new level of individuality. Despite the potential for community-level interactions, most studies have not addressed the competitive context of this transition, such as competition between species. Here, we explore how interspecific competition shapes the emergence of multicellularity in an experimental system with two yeast species, *Saccharomyces cerevisiae* and *Kluyveromyces lactis*, where multicellularity evolves in response to selection for faster settling ability. We find that the multispecies context slows the rate of the transition to multicellularity, and the transition to multicellularity significantly impacts community composition. Multicellular *K. lactis* emerges first and sweeps through populations in monocultures faster than in cocultures with *S. cerevisiae*. Following the transition, the between-species competitive dynamics shift, likely in part to intraspecific cooperation in *K. lactis*. Hence, we document an eco-evolutionary feedback across the transition to multicellularity, underscoring how ecological context is critical for understanding the causes and consequences of innovation. By including two species, we demonstrate that cooperation and competition across several biological scales shapes the origin and persistence of multicellularity.

## 1. Introduction

All life on Earth was shaped by cooperation and competition among individuals. Competition in particular has been emphasized for its role driving diversification and adaptation [1–3]. In nature, several well-known examples like the adaptive radiation of finches on the Galápagos Islands [4] or the global invasions of Mediterranean bay mussels [3] illustrate the significance of these competitive dynamics. In the laboratory, competition among mutants demonstrates striking instances of parallelism and stochasticity, as high-fitness mutations sweep through replicate populations of *Escherichia coli* [5,6]. Nevertheless, several decades of work have also brought greater recognition to the ubiquity of cooperation among evolving populations [7–9], with the interplay of cooperation and conflict facilitating diversification events [10,11]. Since cooperative behaviour has been shown to provide competitive advantages against non-cooperators [12,13]—and cooperation often occurs among individuals also in competition [14,15]—it is clear that the two do not occur in isolation. Both cooperation and competition play important roles in natural systems [16–18], and experimental studies have been key to disentangling their relative influences [19–21].

The most pivotal moments of cooperation among groups are considered Major Evolutionary Transitions (METs) [22,23], more specifically referred to as Evolutionary Transitions in Individuality [24,25], where separately replicating units (e.g. single cells) formed new levels of individuality (e.g. multicellular organisms). When first described, the primary challenge for maintaining cooperative benefits across METs was thought to be the potential for genetic conflict within groups [26]. A central question was therefore, whether mechanisms for suppressing within-group conflict could invade when rare [22,27,28]. Recent advances, however, have demonstrated the significance of cooperation and competition not only within but between groups, with the competitive benefits of group cooperation rapidly realized experimentally [29–31]. These results indicate that in order to understand the emergence of within-group cooperation across METs, the potential for cooperation and conflict between groups also needs to be explored. In nature, competition among species is central to understanding evolutionary outcomes, and interspecific interactions have shaped the evolution of cooperation in many natural systems [17]. Thus far, the vast majority of experiments examining METs involve a single species [32–36], constraining our ability to explore the role of ecological context during the evolution of innovation. Analysis of eco-evolutionary dynamics across METs thus requires a system that incorporates these biological scales. How does competition between species shape the emergence of cooperative groups?

The origin of multicellularity paved the way for adaptations of complex structures and functions and has been readily observed in the laboratory [32,37]. In particular, parallel kinds of multicellularity evolve in two different yeast species: *Saccharomyces cerevisiae* and *Kluyveromyces lactis*, in response to selection for settling through liquid media [29,38]. Despite having diverged phylogenetically approximately 100 million years ago (Ma), these distinct species evolved multicellularity via the same mechanism (‘staying together’ of daughter cells with mother cells) [38,39]. Unlike *S. cerevisiae*, however, *K. lactis* also cooperated with non-relatives to form even larger clusters via floccing [29]. This cooperative behaviour was observed between multicellular clusters, as well as through the attachment of unicellular ‘free riders’ to multicellular neighbours. Since floccing between groups likely provides further competitive advantages when settling, it is clear that key parameters facilitating the transition to multicellularity go beyond the suppression of within-group conflict. Rather, the emergence of multicellularity may depend on competitive benefits conferred via cooperation at the cellular, individual and community levels.

To explore the interplay of cooperation and competition across the transition to multicellularity, we evolved cocultures of *S. cerevisiae* and *K. lactis* with settling selection in separate experiments, starting with either unicellular or multicellular ancestors, i.e. beginning before and after both species had transitioned to multicellularity. Since species are rarely isolated in nature, interspecific competition during the emergence of biological innovation could be critical for claims about transitions in complexity. In particular, competition between species has important adaptive consequences, with evolutionary trajectories dependent on population densities [40,41], the tempo of adaptation [33,34] and competitive ability for essential resources [42]. Thus, in this system, we explored how interspecific competition influences the transition to multicellularity, as well as how the transition shifts the competitive ability among species. We found that the transition to multicellularity dramatically affected evolutionary outcomes, with *K. lactis* emerging first and sweeping through the populations in cocultures. The presence of both species significantly affected this transition, and the nature of interspecific interactions shifted once multicellular. Multicellular responses to selection arise from competition for settling ability both within and between species, in addition to any pre-existing competitive and cooperative interactions. Together, this work documents an eco-evolutionary feedback across the transition to multicellularity, where interactions among species shape the emergence of cooperative groups, which in turn shapes interactions between species. We expand on single-species work, demonstrating how ecological context is critical for understanding transitions in individuality.

## 2. Material and methods

### (a) Strains and media

The unicellular isolates used were *S. cerevisiae* strain Y55 and *K. lactis* strain NRLY-1140, both referred to as ‘ancestors’ in this study. Cultures were grown in 10 ml Yeast Peptone Dextrose media (YPD; 1% yeast extract, 2% peptone, 2% d-glucose) in 25 mm × 150 mm glass culture tubes, at 30°C and shaking at 250 r.p.m. Solid plates were prepared using Yeast Peptone Lactose media (YPL; 1% yeast extract, 2% peptone, 2% lactose, 1.5% agar), which we developed through pilot testing to allow differentiation between species. Clonal populations were established from single colonies.

### (b) Characterization of populations

**Figure 1.**
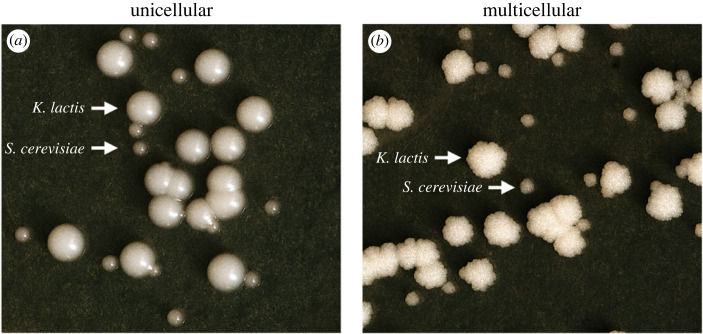
Unicellular (*a*) and multicellular (*b*) colonies of *K. lactis* and *S. cerevisiae* on YPL plates. Multicellularity is easily distinguished by the formation of rugose, rather than smooth, colonies. Species are distinguished by the formation of large *K. lactis* colonies and small *S. cerevisiae* colonies, following 48 h of growth at 30°C.

Standard dilution plating techniques were used to measure colony forming units (CFUs) for each population. To distinguish between species, plating on YPL media allowed *K. lactis* to form larger colonies than *S. cerevisiae*, which were easily identifiable after 48 h of growth at 30°C. The presence of multicellular phenotypes was determined based on colony morphology (smooth unicellular colonies and rugose multicellular colonies; figure 1).

### (c) Settling selection experiments

#### (i) Unicellular ancestors

To evaluate the effects of interspecific competition on the transition to multicellularity, we performed a selection experiment with cocultures of both unicellular species at three different initial frequencies: 25%, 50% and 75%, as well as monocultures of *K. lactis* and *S. cerevisiae*, separately. Six initially isogenic replicate populations per treatment were propagated with benchtop settling selection for 19 days. Every 24 h, 1.0 ml aliquots of well-mixed culture were transferred to 1.5 ml microfuge tubes, where they sat undisturbed for 7 min. The bottom 100 µl was then carefully transferred to 10 ml of fresh YPD media. Six replicate control populations per treatment were propagated via standard daily transfers (without settling). Relative frequencies of each species and morphotype (smooth versus rugose colonies) were measured via dilution plating on YPL plates before 24 h of growth (Day 0), daily for the first three transfers, and then every other day until transfer 19. Ancestral strains, as well as 700 µl subsamples of each population following transfers 10 and 19, were preserved with 25% glycerol and stored at – 80°C.

#### (ii) Multicellular invasion-from-rare

Since *S. cerevisiae* remained unicellular in cocultures during the above experiment, we conducted a separate experiment beginning with multicellular isolates to assess whether each species could invade when rare following the transition to multicellularity. Six replicate populations per treatment (5% initial frequency *K. lactis* and 5% initial frequency *S. cerevisiae*) were transferred every 24 h with and without settling selection for 20 days. YPL plates were inoculated for each population after 24 h of growth and after 10 and 20 transfers. Subsamples were preserved every 10 days with 25% glycerol at –80°C. The change in the relative proportion of the two competing multicellular species was measured as the differential growth rate (*s*) [43]:

$$s=\ln\left[\frac{x_{1}(t)}{x_{2}(t)}\right],$$

where *x*_1_(*t*) and *x*_2_(*t*) represent the relative frequency of *S. cerevisiae* and *K. lactis* by timepoint (*t*), respectively.

#### (iii) Selection coefficients

To assess the rate that multicellular *K. lactis* swept through the population during settling selection with unicellular ancestors, we calculated the differential growth rate as the natural log of the frequency of multicellular *K. lactis* (*x*_1_) over the frequency of all other strains (*x*_2_) for each replicate population, beginning on the first day multicellular *K. lactis* emerged and ending when the population appeared to plateau (see above). We estimated the slope (selection coefficient) by linear regression of differential growth rate over these times and compared the mean selection coefficients across treatments.

### (d) Competition assays

To determine competitive dynamics across the MET, we performed a series of competition assays with unicellular and multicellular strains of each species. To assess competitive growth ability between species without settling, we inoculated 10 ml of YPD with 5 µl of ancestral *K. lactis* and 95 µl of ancestral *S. cerevisiae* (and *vice versa*), establishing six replicate populations when each species begins as rare. CFUs were measured via dilution plating before and after 24 h of growth. To assess competitive settling ability, 10 ml of YPD were inoculated with 5 µl of one species and 95 µl of the other (all unicellular, six replicates each) and grown overnight. CFUs were then measured with dilution plating before and after one round of settling (bottom 100 µl of 1.0 ml subculture transferred following 7 min of benchtop settling). All of the above procedures were repeated for multicellular strains to determine competitive growth and settling abilities before and after the transition to multicellularity.

Forty-eight hour growth curve data were acquired for unicellular isolates using a Tecan infinite 200pro microplate reader. We transferred 5 µl of overnight cultures to 195 µl of YPD in 96-well plates, with 10 replicates per treatment: monocultures of each species and cocultures of 50% each. Growth was measured by OD600 absorbance reads every 15 min. Population-level information like carrying capacity (*K*) and intrinsic growth rate (*r*) were determined by fitting growth curve data to the logistic equation for population size *N_t_* at time *t*:

$$N_{t}=\frac{K}{1+((K-N_{0})/N_{0})e^{-rt}},$$

where *N*_0_ is the population size at the beginning of the growth curve (R package Growthcurver) [44].

To assess the possibility that each species could use a by-product of the other, we grew *K. lactis* and *S. cerevisiae* on their own and each other's spent media. To do this, we inoculated 10 ml of YPD with 100 µl of a grown culture of each species, separately, and grew the monocultures for 24 h (15 tubes per species). Following overnight growth, we removed the cells using a sterile 0.22 µl filter, combining the spent media from each replicate into sterile glass bottles, and then distributing 10 ml into fresh tubes. Each tube was inoculated with 100 µl of overnight cultures, with six replicates per treatment (four treatments: each species grown on its own and each other's spent media). After 24 h of growth on spent media, density of cells was measured via dilution plating and counting CFUs.

## 3. Results

### (a) *Kluyveromyces lactis* becomes multicellular first in monocultures

**Figure 2.**
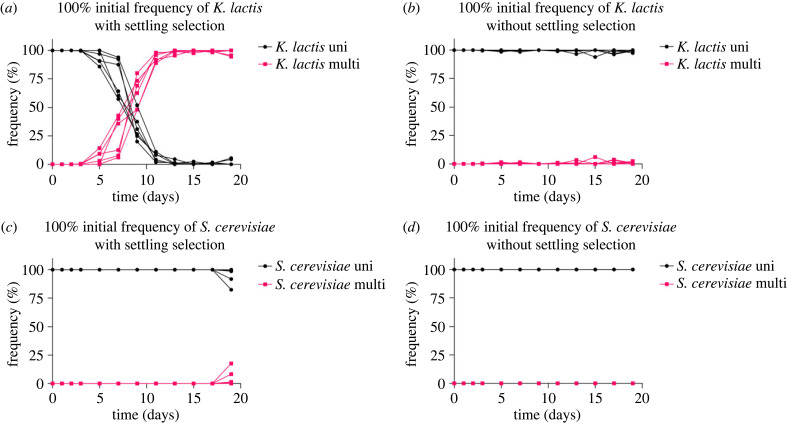
Relative frequencies of unicellular and multicellular *K. lactis* and *S. cerevisiae* in monocultures during 19 days with and without settling selection. Monocultures began as isogenic unicellular isolates with an initial frequency of 100% *K. lactis* or *S. cerevisiae* (six replicate populations per treatment). Over the course of the experiment, multicellular *K. lactis* emerged and increased in frequency when propagated with settling (*a*) but not without settling selection (*b*). Monocultures of *S. cerevisiae* remained unicellular until day 19 with settling selection (*c*) and never evolved multicellularity in control replicates (*d*).

Monocultures of each unicellular species were propagated with and without settling selection for 19 days. When transferred with settling, multicellularity in *K. lactis* emerged first, with all six replicate populations containing multicellular individuals after 7 days of selection (figure 2*a*). In the *S. cerevisiae* monocultures, however, multicellularity did not appear until day 19 of the experiment (figure 2*c*) and was never observed without settling (figure 2*d*). The occasional appearance of multicellular colonies even in the *K. lactis* control populations (figure 2*b*) suggests that the mutation causing multicellular clusters either occurs more frequently than in populations of *S. cerevisiae*, or its effects are masked in *S. cerevisiae* heterozygotes [37].

### (b) Species coexist in the absence of settling

**Figure 3.**
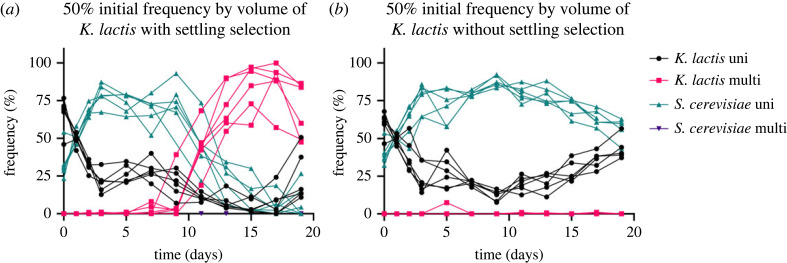
Relative frequencies of *K. lactis* and *S. cerevisiae* in cocultures during 19 days with and without settling selection. Cocultures began as isogenic unicellular isolates with an initial frequency of approximately 50% *K. lactis* (volumetric). Unicellular *S. cerevisiae* remains at a higher frequency than unicellular *K. lactis* until multicellular *K. lactis* emerges during settling selection (*a*). *S. cerevisiae* appears to be excluded in three out of the six experimental replicates (*a*), whereas species coexist in all control populations (*b*) (see electronic supplementary materials for cocultures with initial frequencies of 25% and 75% *K. lactis*; electronic supplementary material, figures S1 and S2).

**Figure 4.**
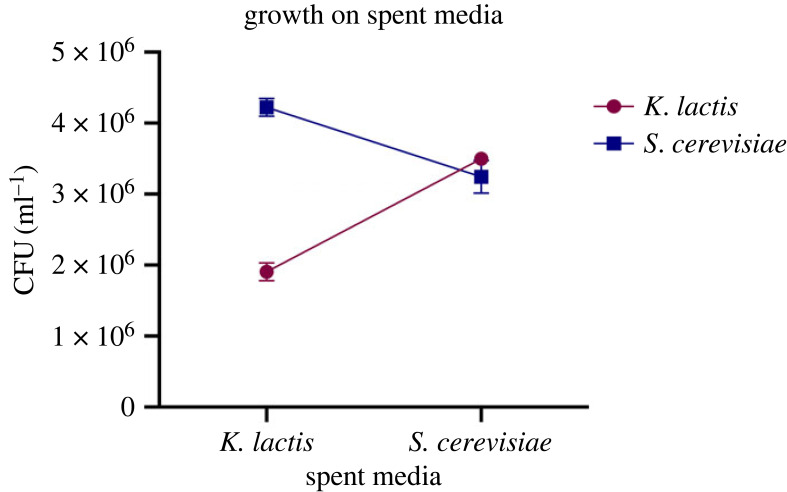
Density of cells after 24 h of growth on spent media. *K. lactis* reached a greater density when grown on spent media from *S. cerevisiae*, compared with its own spent media (*p* < 0.0001, one-way ANOVA, electronic supplementary material, table S2). Likewise, there was a greater density of *S. cerevisiae* following growth on *K. lactis* spent media, compared with its own (*p* = 0.0041, one-way ANOVA; electronic supplementary material, table S3). Results indicate that the two species occupy different niches in YPD media, possibly due to their ability to grow on a by-product of the other.

Without settling selection, species remained unicellular and coexisted throughout the 19 day experiment in cocultures (figure 3*b*; electronic supplementary material, figure S1). To understand why they could coexist, we grew each species on the other's spent media (see Material and methods) and found that both species reached a greater density when grown on the spent media of the other species, compared with their own (figure 4) (*K. lactis*: *p* < 0.001, *S. cerevisiae*: *p* = 0.0041, one-way ANOVA; electronic supplementary material, tables S1 and S2). This indicates that both species may be able to grow on a by-product of the other, or otherwise preferentially use different resources in the media. The species therefore occupy sufficiently different niches when grown together in YPD media, enabling their coexistence.

### (c) Multispecies context slows the emergence of multicellularity

As expected from the above results in monocultures, multicellular *K. lactis* appeared first in cocultures with *S. cerevisiae* and rapidly rose in frequency during 19 days of settling selection (figure 3*a*; see electronic supplementary materials for cocultures with initial frequencies of 25% and 75% *K. lactis*; electronic supplementary material, figure S2). In some cases, multicellular *K. lactis* ultimately appeared to exclude *S. cerevisiae*; there were no *S. cerevisiae* colonies present per approximately 100 CFUs after 19 days in three out of six replicate populations when *K. lactis* had an initial frequency of 25% and 50%, and *S. cerevisiae* was absent in four out of six populations when *K. lactis* had an initial frequency of 75%. This suggests that a different tempo in the transition to multicellularity may lead to competitive exclusion. Unicellular *K. lactis*, however, was retained in the populations and sometimes increased in frequency at the end of the experiment. This is likely due to the adaptation of unicellular free-riders, which have been found to evolve a greater competitive ability, compared with unicellular ancestors, in the *K. lactis* species [29].

**Figure 5.**
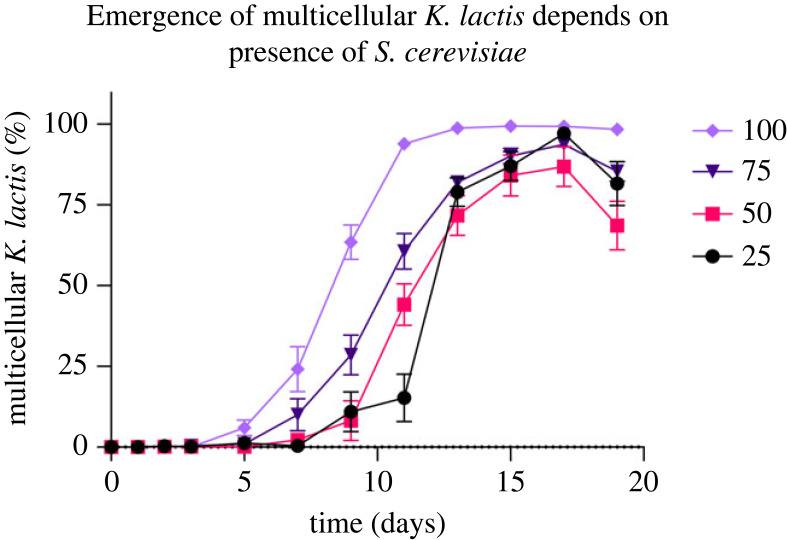
Frequency of *K. lactis* multicellular clusters during 19 days of settling selection, with initial unicellular *K. lactis* frequencies of 25%, 50%, 75% and 100% (error bars ± s.e.m.). Tempo of the transition to multicellularity depends on time and the presence of *S. cerevisiae* (*adj. r*^2^ = 0.67, *p* < 0.0001 (for both factors), linear regression).

The rate that multicellular *K. lactis* swept through the populations differed between treatments (figure 5). We calculated the selection coefficients during the sweep of multicellular *K. lactis* (see Material and methods) and found that populations without *S. cerevisiae* (100% *K. lactis*) had a greater mean selection coefficient, compared with cocultures with initial frequencies of 75% (*p* = 0.0086, Tukey–Kramer honest significant difference (HSD); electronic supplementary material, table S3), 50% (*p* = 0.0115) and 25% *K. lactis* (*p* = 0.0234) (table 1). There were no significant differences in the mean selection coefficients among cocultures, when initial frequency was treated as categorical (*p* > 0.05, Tukey–Kramer HSD), nor when it was continuous (*p* > 0.05, linear regression). Results indicate that the rate of the multicellular *K. lactis* sweep depends on the presence of *S. cerevisiae*; therefore, interspecies interactions shaped the response to selection.

**Table 1.** Selection coefficients during multicellular sweep of *K. lactis* for each initial frequency. Coefficients determined from the slope of each linear regression for the differential growth rate for each replicate (six replicates per treatment). Populations without *S. cerevisiae* (100% initial frequency *K. lactis*) have greater selection coefficients during the increase in frequency of multicellular *K. lactis*, compared with cocultures with an initial frequency of 75% (*p* = 0.0086, Tukey–Kramer HSD; electronic supplementary material, table S1), 50% (*p* = 0.0115) and 25% (*p* = 0.0234). Asterisk indicates a significant difference among groups, with a *p* value < 0.05.

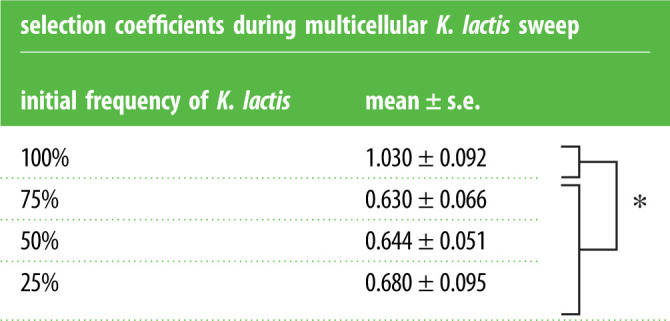

### (d) Multicellular invasion is species specific

**Figure 6.**
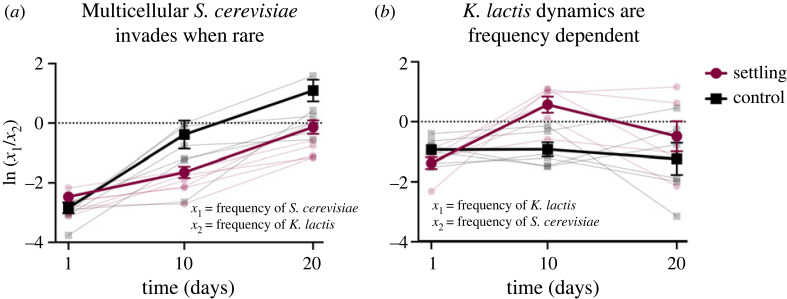
Differential growth rate of multicellular *K. lactis* and *S. cerevisiae* after 1, 10 and 20 days of selection, when (*a*) *S. cerevisiae* and (*b*) *K. lactis* begin as rare. When *S. cerevisiae* is rare, differential growth rate depends on time (adj. *r*^2^ = 0.0812, *p* < 0.0001, linear regression; electronic supplementary material, table S4) and settling condition (*p* = 0.032), with the effect of condition changing over time (*p* = 0.0005). When *K. lactis* is rare, differential growth rate depends on time (adj. *r*^2^ = 0.445, *p* < 0.0001, linear regression; electronic supplementary material, table S5) and settling condition (*p* = 0.0233), with an interaction between condition and time (*p* = 0.0086). Bold lines indicate the mean for each treatment (error bars ± s.e.m.), with fainter lines indicating individual replicates.

We performed a 20 day invasion-from-rare experiment beginning with multicellular isolates of both species, since *S. cerevisiae* did not become multicellular during 19 days of settling selection with *K. lactis*. We found that when both species began as multicellular, the dynamics of their invasion was species specific. When *S. cerevisiae* began as rare, it initially only increased in frequency when grown without settling but later appeared to evolve greater competitive ability when settling, allowing both treatments to increase in frequency after 20 days (figure 6). Thus, differential growth rate between the species depends on time (adj. *r*^2^ = 0.824, *p* < 0.0001, linear regression; electronic supplementary material, table S4) and settling condition (*p* = 0.032), with the effect of condition changing over time (*p* = 0.0005). When *K. lactis* began as rare, it initially invaded when settling, but neither condition continued to increase in frequency between 10 and 20 days, demonstrating frequency-dependent selection. The differential growth rate in this case depends on time (adj. *r*^2^ = 0.445, *p* = 0 < 0.0001, linear regression; electronic supplementary material, table S5) and settling condition (*p* = 0.023), with an interaction between condition and time (*p* = 0.0086). To determine whether these invasion dynamics differed by species, we compared the mean selection coefficients (i.e. the slope of the differential growth rate over time) between conditions when *S. cerevisiae* versus *K. lactis* was rare, for control and settling populations, during the first and final 10 days of the experiment. We found that the control populations were significantly different both during the first 10 days (*p* = 0.0039, *t*-test, electronic supplementary material, table S6) and final 10 days (*p* = 0.0380, *t*-test, electronic supplementary material, table S6), whereas the settling populations did not differ significantly during the first 10 days (*p* = 0.9310, *t*-test, electronic supplementary material, table S6) but did between days 10 and 20 (*p* = 0.0010, *t*-test; electronic supplementary material, table S6), indicating that population dynamics depend on which species begins as rare. Thus, when the tempo of the transition to multicellularity is similar such that both species are multicellular, the species are able to coexist, but the nature of their invasion is species specific.

### (e) Competitive dynamics shift across the transition to multicellularity

We obtained 48 h growth curves to assess the competitive dynamics between species before the transition to multicellularity and found that monocultures of *S. cerevisiae* had the highest population growth rates (*r*) (*r* = 0.808 ± 0.009 s.e.), followed by the cocultures (*r* = 0.749 ± 0.004 s.e.), and monocultures of *K. lactis* (*r* = 0.547 ± 0.003 s.e.), with a significant difference among the three treatments (*p* < 0.0001, one-way ANOVA; electronic supplementary material, table S7) (electronic supplementary material, figure S3).

**Figure 7.**
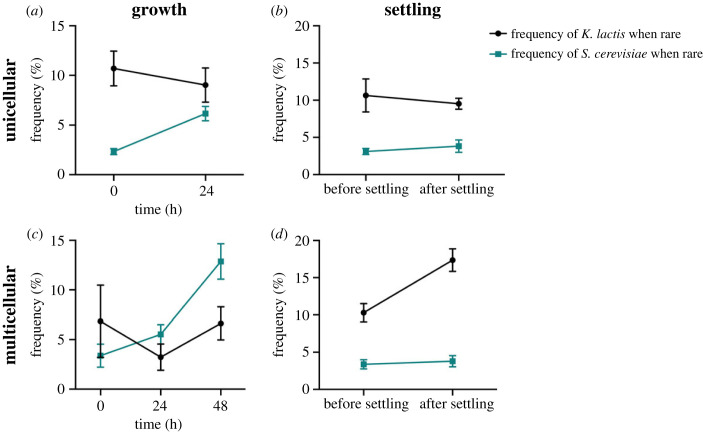
Invasion dynamics of *S. cerevisiae* and *K. lactis*. Frequency of each species, grown together without settling when unicellular (*a*) and multicellular (*c*), and before and after settling when unicellular (*b*) and multicellular (*d*), when either *K. lactis* or *S. cerevisiae* begin as rare. Relative frequency of *S. cerevisiae* increases from rare after 24 h of growth when unicellular (*p* = 0.0006, linear regression) but only after 48 h of growth when multicellular (*p* = 0.0013, linear regression), whereas frequency of *K. lactis* when rare does not change in both conditions (unicellular: *p* = 0.51, linear regression; multicellular: *p* = 0.37, linear regression). Relative frequencies of unicellular *K. lactis* and *S. cerevisiae* do not change after 7 min of settling, when either species begins as rare (*S. cerevisiae* rare: *p* = 0.45, linear regression; *K. lactis* rare: *p* = 0.64, linear regression). When multicellular, relative frequency of *K. lactis* increases after 7 min of settling (*p* = 0.0047, linear regression), whereas frequency of *S. cerevisiae* does not change significantly when rare (*p* = 0.68, linear regression). Multicellular *K. lactis* has a competitive advantage when settling—an advantage that was not present when both species were unicellular. Initial frequency of *K. lactis* is greater due to smaller cell size and therefore more cells per millilitre (5 µl inoculation when rare).

This finding aligns well with results from competition assays (figure 7), where the relative frequency of *S. cerevisiae* increased from rare after 24 h of growth when unicellular (*p* = 0.0006, linear regression; electronic supplementary material, table S8), but the frequency of *K. lactis* when rare did not change (*p* = 0.51, linear regression; electronic supplementary material, table S9). Following 7 min of settling, however, there was no significant change in frequency from rare for either species (*S. cerevisiae* rare: *p* = 0.45, linear regression; electronic supplementary material, table S10; *K. lactis* rare: *p* = 0.64, linear regression; electronic supplementary material, table S11). Results indicate that *S. cerevisiae* has a competitive advantage when grown together (figure 7*a*), but neither species can invade as a result of settling alone (figure 7*b*), when unicellular.

These competitive dynamics shift once the strains are multicellular. In the absence of settling, the increase in frequency of *S. cerevisiae* when grown together was no longer significant after 24 h (*p* = 0.19, linear regression; electronic supplementary material, table S12), although it was after 48 h (*p* = 0.0013, linear regression; electronic supplementary material, table S13), when multicellular. Similar to when unicellular, the frequency of *K. lactis* did not change significantly after 24 h (*p* = 0.37, linear regression; electronic supplementary material, table S14) or 48 h (*p* = 0.96, linear regression; electronic supplementary material, table S15) of growth (figure 7*c*). When settling, however, multicellular *K. lactis* increased significantly from rare (*p* = 0.0047, linear regression; electronic supplementary material, table S16), while the frequency of *S. cerevisiae* remained relatively unchanged (*p* = 0.68, linear regression; electronic supplementary material, table S17) (figure 7*d*). Thus, *S. cerevisiae* still appears to have a growth advantage when multicellular, but multicellularity provides a competitive settling advantage for *K. lactis* that was not present when unicellular.

## 4. Discussion

This work documents how ecological context shapes the evolution of multicellularity, which in turn shapes the ecological context of two species. We expand on previous findings in a single-species system, where sociality emerged and strengthened the benefits of multicellularity in *K. lactis*, underscoring the importance of ecological context across the transition. By incorporating both *K. lactis* and *S. cerevisiae*, we demonstrate not only the significance of cooperative and competitive dynamics across several scales but also how transitions in complexity can themselves be understood as eco-evolutionary feedbacks.

### (a) Ecological context shapes evolution of multicellularity

When unicellular, we found that *S. cerevisiae* had a numerical advantage when grown with *K. lactis*. This was demonstrated both by their difference in growth rates and competition assays, where only *S. cerevisiae* could invade when rare. Nevertheless, the two species could coexist over evolutionary time, as observed in our control populations. Once multicellularity emerged, however, the rate at which multicellular *K. lactis* swept through the populations slowed when both species were initially present. This delay in the emergence of multicellularity may be owed to the greater competitive ability of *S. cerevisiae* when grown together, despite its disadvantage when settling. However, since interactions among species can include both negative (e.g. competitive) and positive components (e.g. as shown via faster growth on the other species' spent media), we can only infer that the sum of these interactions with *S. cerevisiae* slowed the transition in *K. lactis.* In addition to shaping the selection coefficients of multicellular *K. lactis*, the presence of *S. cerevisiae* also appeared to increase the rate at which unicellular competitive ability evolved, as more unicellular *K. lactis* are present in cocultures at the end of the experiment, compared with *K. lactis* alone. Hence, the emergence of multicellularity and sociality in one species may depend on interactions with the other.

### (b) Evolution of multicellularity shapes ecological context

As a consequence of multicellularity arising in *K. lactis*, *S. cerevisiae* either became rare or appeared to be excluded from the populations. In the cases of competitive exclusion, this evolutionary outcome appears to mark a dramatic shift in the ecological landscape, where the species had previously coexisted. Exclusion also precludes the emergence of multicellularity in *S. cerevisiae*. In the instances where both species persist, *S. cerevisiae* becomes rare as a result of competition with multicellular *K. lactis*, which presumably would allow multicellularity to arise concurrently in both species. Even so, the tempo of the transition to multicellularity would likely be prolonged in *S. cerevisiae*, as the population would be smaller. Since multicellular invasion is species specific, we demonstrate how the tempo of this transition shapes community composition, an example of priority effects during the emergence of multicellularity.

The evolution of multicellularity also shifts the competitive dynamics between species. Where neither species demonstrated a settling advantage when unicellular, only *K. lactis* can invade from rare when both are multicellular. This ecological shift agrees with previous findings that floccing among multicellular clusters provides a greater settling ability for *K. lactis* [29]. Here, we document the evolutionary consequences of this intraspecific cooperation.

In this experiment, the unicellular species compete for resources and in some cases cooperate perhaps through by-product utilization. Our selection regime then introduces a second level of competition, which shifts the competitive environment: competition for settling ability. Since *K. lactis* is able to respond to this selection pressure faster, settling selection provides a benefit to *K. lactis* and alters the interaction landscape.

### (c) Dynamics of adaptation and diversification

Disentangling how life adapts and diversifies is central to understanding both the history of life on Earth and predicting its future. Experimental evolution has been an important tool for exploring processes of adaptation and diversification, in particular among microbial taxa [45]. The Long-Term Evolution Experiment (LTEE) began in large part to explore these dynamics, and over thousands of generations, has challenged previous ideas that sustained adaptation usually only occurs in response to environmental change. Instead, it appears that while the rate of fitness gain declines over time, both adaptation and divergence can continue unbounded, even in a constant environment [46]. Adaptation even led to the emergence of a significant metabolic innovation in 1 out of the 12 LTEE lines, where *E. coli* gained the ability to use citrate [47]. Interest in the lack of an ‘upper limit’ to adaptation has also led to an increasing recognition for the role that niche construction plays during the diversification of species [48]. As organisms modify niches, they shift selective pressures as well, leading to the convergence of ecological and evolutionary timescales and eco-evolutionary feedbacks [49]. These feedbacks have been well-studied over short timescales in single-species systems, where the effects of constructed environments depends on the abundance of organisms (e.g. frequency and density-dependent selection) [40,41]. Even rapid diversification events like adaptive radiations have been observed in single-species experiments, such as the emergence of niche specialists in *Pseudomonas fluorescens*, where negative frequency-dependent selection maintains distinct morphologies [10].

More recently, the importance of interspecific interactions in shaping eco-evolutionary dynamics has led to a large number of multispecies experimental studies [49–54]. By involving several species, these studies have demonstrated a positive feedback between microbial diversity, the construction of new ecological niches and further diversification [50,55], as well as the coexistence of several species via intransitive interactions [56]. In the work presented here, we observe species coexistence both prior to the emergence of multicellularity and when both species begin as multicellular. The dynamics in these two scenarios meet expectations where species coexist either by occupying different niches, or benefitting from niche construction of the other, as shown by growing each species on spent media. It is the emergence of multicellularity, however, that shifts these dynamics. Our findings therefore contribute to a less-explored area of the literature: the role of eco-evolutionary feedbacks during the emergence of biological innovation [14,48,57]. In our system, the appearance of multicellularity in *K. lactis* dramatically shifts interspecific interactions such that in several cases, *S. cerevisiae* is excluded. As a consequence, we observe divergent outcomes, whereby concurrent emergence of multicellularity in both species depends on historical contingencies.

Additionally, the dynamics in our system do not appear to stabilize over time. As multicellularity and sociality emerge in *K. lactis*, new evolutionary strategies emerge as well, such as increased competitive ability in unicellular *K. lactis*, and the evolution of greater settling ability of multicellular *S. cerevisiae* when in competition with multicellular *K. lactis*. As a result, eco-evolutionary feedbacks seem to continue following this MET, possibly promoting further adaptation, divergence and innovation.

Previous literature on METs has been careful to caution that the adaptive benefits provided by these transitions cannot be used as an explanation for their origin [22]. Nevertheless, it can be easy for retrospective explanations to strip away the ecological complexity that must have been present. In this paper, we show that just one step in ecological complexity (i.e. two species instead of one) has profound consequences for how multicellularity emerges.

### (d) Evolution of complexity

When the concept of METs was first proposed, a primary concern was the potential for within-group conflict to inhibit cooperation [22,26]. In our system, this risk is mitigated since multicellularity arises via ‘staying together’ of cells during asexual reproduction, a possibility previously recognized when a multicellular individual is clonal [58–60]. Instead, our work highlights the potential for cooperation both within and between groups, as well as competition not only among unicellular ancestors but with other species in the community. Elements of determinism and stochasticity found here contribute to findings from other parallel replay experiments [5], as well as the evolution of complexity more generally. While METs have long been recognized for their ability to transform adaptive landscapes, how the landscape transforms the transition itself—and its corresponding feedback—is key to understanding the evolution of complexity.

## Data Availability

All raw data presented in this paper is archived on Zenodo (Data set): https://doi.org/10.5281/zenodo.8263618 [61]. The data are provided in electronic supplementary material [62].
